# Association of psychosis with cognitive impairment is mediated by amyloidopathy in cognitive impairment

**DOI:** 10.3389/fnagi.2025.1663120

**Published:** 2026-01-12

**Authors:** Jung-Min Pyun, Sungjoo Han, Sang Won Park, Na Young Yeo, Young Ho Park, Sang Yun Kim, Young Chul Youn, Jae-Won Jang, and the Alzheimer’s Disease Neuroimaging Initiative

**Affiliations:** 1 Department of Neurology, Seoul National University Bundang Hospital and Seoul National University College of Medicine, Seongnam, Republic of Korea; 2 Department of Neurology, Soonchunhyang University Seoul Hospital, Soonchunhyang University College of Medicine, Seoul, Republic of Korea; 3 Department of Next Generation Big Data Center, Kangwon National University Hospital, Chuncheon, Republic of Korea; 4 Department of Neurology, Kangwon National University Hospital, Kangwon National University College of Medicine, Chuncheon, Republic of Korea; 5 Department of Medical Bigdata Convergence, Kangwon National University, Chuncheon, Republic of Korea; 6 Department of Neurology, Chung-Ang University Hospital, Seoul, Republic of Korea

**Keywords:** Alzheimer’s disease, amyloidopathy, cerebrospinal fluid, cognition, mediation, neurodegeneration, psychosis, tauopathy

## Abstract

**Background:**

Psychosis, including delusions and hallucinations, is a significant neuropsychiatric symptom in Alzheimer’s disease (AD) associated with poor prognosis. The relationship between psychosis and AD pathology remains controversial. This study investigates the role of AD pathology in mediating the association between psychosis and cognitive impairment.

**Methods:**

Data were obtained from the Alzheimer’s Disease Neuroimaging Initiative (ADNI). We included individuals with a Clinical Dementia Rating (CDR) score of 0.5 or higher. Among a total of 833 individuals, 96 individuals with psychosis were matched to 192 individuals without psychosis using propensity scores based on age, sex, education level, and follow-up duration. Baseline cognitive performance was assessed using composite memory scores (ADNI-MEM) and executive function scores (ADNI-EF). AD pathology was measured using baseline cerebralspinal fluid (CSF) levels of *β*-amyloid_1-42_ (Aβ_1-42_), hyperphosphorylated-tau_181_ (p-tau_181_), and total tau. Logistic regression was performed to evaluate the association of psychosis with baseline cognitive performance and CSF biomarkers. Mediation analysis was conducted to assess whether AD biomarkers mediate the relationship between cognitive impairment and psychosis.

**Results:**

Psychosis was significantly associated with worse ADNI MEM score (*β* = −0.622, *p* = 0.013) and worse ADNI EF score (*β* = −0.516, *p* = 0.003), and lower CSF Aβ_1-42_ levels (*β* = −0.009, *p* = 0.007). No significant associations were found with p-tau_181_ or total tau levels. Mediation analysis revealed that low CSF Aβ_1-42_ levels mediated the relationship between cognitive impairment and psychosis.

**Conclusion:**

These findings suggest that amyloid pathology may mediate the effect of baseline cognitive impairment on psychosis during disease in AD, highlighting a potential pathological link between cognitive decline and psychotic symptoms.

## 1 Introduction

Neuropsychiatric symptoms (NPS) are frequent in neurodegenerative diseases with the prevalence estimating up to 97% (Steinberg et al., 2008; Cerejeira et al., 2012). NPS are present in the majority of Alzheimer’s disease (AD) dementia, and have recently been highlighted as an early sign of AD in the preclinical stage (Naasan et al., 2021; Goukasian et al., 2019; Eikelboom et al., 2021; Lussier et al., 2020). Among NPS, psychosis consists of delusions and hallucinations, and is a clinically significant symptom that causes severe distress to caregivers (Connors et al., 2018). Moreover, psychosis in AD is associated with poor prognosis including rapid cognitive and functional decline, earlier institutionalization, and increased mortality (Scarmeas et al., 2005; Peters et al., 2015; Fischer et al., 2012; Toot et al., 2017).

Despite its clinical significance, the underlying pathomechanism of psychosis in AD has not yet been fully elucidated. Although the relationship between psychosis and various AD-related pathologies, including amyloidopathy and tauopathy has been investigated, the findings remain inconclusive. An association between psychosis and an increased load of neuritic plaques has been reported in pathologically confirmed AD cases without cognitive impairment (Kim et al., 2018). Post-mortem analyses have demonstrated that an increased burden of neurofibrillary tangles (Zubenko et al., 1991; Förstl et al., 1994; Farber et al., 2000) and phosphorylated tau (Förstl et al., 1994; Farber et al., 2000; Murray et al., 2014; Koppel et al., 2014) is associated with psychosis. Studies using AD biomarkers have reported that cognitive decline in AD patients with psychosis correlates with increased tau retention on positron emission tomography (PET) and elevated cerebrospinal fluid (CSF) total tau levels (Koppel et al., 2013). Association of plasma hyperphosphorylated-tau_181_ (p-tau_181_) with emergence of psychosis in AD have been reported (Gomar and Koppel, 2024).

The incidence of psychosis increases during AD progression from 20% in 1-year to 51% in 4-year after AD diagnosis (Paulsen et al., 2000). The factors related to incidence of psychosis included severity of cognitive impairment and the rate of cognitive decline (Paulsen et al., 2000; Weamer et al., 2009; Weamer et al., 2016). However, the underlying pathological mechanism of poor cognitive function leading increased psychosis during AD progression is not clear. We wondered whether psychosis arises primarily from impaired perception and information processing associated with worsening cognition, or whether it is linked to molecular AD pathology.

To address this question, we selected patients with mild cognitive impairment (MCI) and dementia from the ADNI cohort. We investigated the association between the presence of psychosis during longitudinal follow-up and baseline cognitive function, as well as baseline CSF AD biomarkers, including Aβ_1-42_, p-tau_181_, and total tau (t-tau). We then investigated whether CSF AD biomarkers mediated the relationship between baseline cognitive function and the presence of psychosis. Finally, we explored the association between psychosis during the disease course and longitudinal changes in CSF AD biomarkers.

## 2 Methods

### 2.1 Participants

Data used in this study were obtained from the Alzheimer’s Disease Neuroimaging Initiative (ADNI) database (adni.loni.usc.edu). The ADNI was launched in 2003 as a public-private partnership. The primary goal of ADNI has been to test whether serial magnetic resonance imaging, PET, other biological markers, and clinical neuropsychological assessment can be combined to measure the progression of MCI and early AD (Aisen et al., 2015). We included participants with MCI and AD dementia, who underwent neuropsychological assessment with Neuropsychiatric Inventory-Questionnaire (NPI-Q) (Cummings et al., 1994). We selected participants with a clinical dementia rating (CDR) (Morris, 1993) score of 0.5 and higher.

Presence or absence of psychosis was assessed using the first two items (delusions and hallucinations) of the 12-item NPI-Q. Participants were classified as having psychosis (Psychosis) if they exhibited delusions or hallucinations at any visit during follow-ups, and as no-psychosis if neither symptom was reported at any visit (No-psychosis) (Gomar et al., 2022). The baseline psychosis assessment was aligned with the baseline CDR evaluation, and participants with psychosis at baseline were included in the analysis.

### 2.2 CSF measurement

For estimation of amyloid, tau, and neurodegenerative burden, the concentrations of baseline CSF Aβ_1-42_, p-tau_181_, and t-tau were used, respectively. CSF biomarkers were measured by a microbead-based multiplex immunoassay (INNO-BIA AlzBio3 RUO test; Fujirebio, Ghent, Belgium) (Shaw et al., 2009). Details of CSF collection are explained in the ADNI website.^1^

### 2.3 Cognitive assessment

Cognitive function was assessed using mini-mental state examination (MMSE) (Folstein et al., 1975), CDR, CDR sum of boxes (CDR SOB), composite scores of memory in ADNI (ADNI MEM) (Crane et al., 2012), and composite score of executive function in ADNI (ADNI EF) (Gibbons et al., 2012).

### 2.4 Statistical analysis

We performed propensity score matching to minimize selection bias between Psychosis and No-psychosis. Propensity scores were calculated using logistic regression (Rosenbaum and Rubin, 1985) with covariates including baseline age, sex, educational level, and follow-up duration, as age and sex represent demographic characteristics, whereas educational level and follow-up duration have been implicated as potential risk factors for psychosis (Ropacki and Jeste, 2005). The analysis was performed using the *MatchIt* package in *R. psychosis* and No-psychosis were paired 1:2 based on these propensity scores with a caliper size of 0.2.

Baseline demographics and clinical characteristics between matched participants were compared with Student’s *t*-test or the chi-squared test. Association of the presence of psychosis during disease and baseline cognitive function assessed by MMSE, ADNI MEM, and ADNI EF were analyzed using logistic regression model adjusted for age, sex, education level, CDR SOB, and *APOE* ε4 carrier status. Associations of the presence of psychosis during disease and baseline CSF Aβ_1-42_, p-tau_181_, and t-tau levels were analyzed using logistic regression model adjusted for age, sex, CDR SOB, and *APOE* ε4 carrier status.

Mediation analysis was performed between baseline cognitive function assessed by ADNI MEM and ADNI EF, and Psychosis using process packages in R. Mediator was CSF Aβ_1-42_, which association with Psychosis was significant. Covariates were age, sex, educational level, and follow-up duration.

The association of psychosis during disease and longitudinal CSF AD biomarker was evaluated using linear mixed model. CSF AD biomarkers assessments were included with follow-up periods of up to 36 months, due to a substantial decreased in the frequency of CSF measurements. The model was adjusted for age, sex, and *APOE* ε4 carrier status. The interaction term (psychosis during disease × time) was assessed.

All statistical analysis was performed using R (version 4.2.3). Statistical significance was set at < 0.05.

## 3 Results

### 3.1 Baseline demographics of study participants

A total of 833 individuals, Psychosis (*n* = 96) and No-psychosis (*n* = 737), were included. After propensity score matching, Psychosis (*n* = 96) and No-psychosis (*n* = 192) were selected for further analysis. After PSM, all variables achieved acceptable balance with absolute standardized mean differences (SMD) < 0.1, except for age (SMD = 0.151). Baseline demographic characteristics of matched participants were shown in Table 1. Among patients in the psychosis group, 12 (12.5%) exhibited psychosis at baseline, and the mean time to psychosis onset during follow-up was 6.0 months (standard deviation = 13.5). Psychosis presented poor cognitive performances than the No-psychosis in MMSE, ADNI MEM, ADNI EF, and poor functional scores in CDR and CDR SOB. In CSF biomarkers, Psychosis group showed lower A*β*_1-42_ level compared to the No-psychosis group.

**Table 1:** Demographics of study participants.

Variables	Psychosis (*n* = 96)	No-psychosis (*n* = 192)	*p*-value
Age	74.5 ± 7.4	75.6 ± 7.9	0.248
Female, n (%)	42 (43.7)	84 (43.7)	1.000
Education level	15.3 ± 3.0	15.3 ± 2.9	0.888
*APOE* ε4 carrier, n (%)	64 (66.6)	95 (49.4)	0.008
Follow-up duration (months)	21.1 ± 15.6	20.1 ± 16.9	0.651
Psychosis at baseline, n (%)	12 (12.5%)	0	NA
MMSE	25.2 ± 2.5	26.5 ± 2.7	<0.001
CDR	0.6 ± 0.2	0.5 ± 0.1	<0.001
CDR SOB	3.5 ± 1.9	2.0 ± 1.6	<0.001
ADNI MEM	−0.6 ± 0.6	−0.1 ± 0.7	<0.001
ADNI EF	−0.7 ± 1.0	−0.07 ± 0.9	<0.001
CSF Aβ_1-42_ (pg/mL)	141.4 ± 36.8	168.9 ± 54.0	<0.001
CSF p-tau_181_ (pg/mL)	41.9 ± 19.9	40.8 ± 23.2	0.687
CSF t-tau (pg/mL)	111.7 ± 54.2	105.5 ± 56.7	0.372

Data are presented as the mean ± standard deviation or n (%). Aβ, β-amyloid; ADNI EF, composite score of executive function in ADNI; ADNI MEM, composite scores of memory in ADNI; CDR, clinical dementia rating; CDR SOB, clinical dementia rating sum of boxes; CSF, cerebrospinal fluid; MMSE, mini-mental state examination; NA: not applicable; p-tau, hyperphosphorylated tau; t-tau, total tau.

We additionally compared baseline demographics between the psychosis and non-psychosis groups after excluding participants with psychosis at baseline (Supplementary Table 1). The group differences were consistent with those observed in analyses including participants with psychosis at baseline.

### 3.2 Association of psychosis with baseline cognitive function

**Figure 1:**
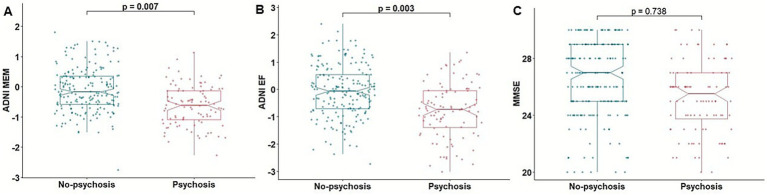
Association of psychosis with cognitive function. Boxplots depicting the association of psychosis with baseline cognitive function assessed by ADNI MEM **(A)**, ADNI EF **(B)**, and MMSE **(C)**. ADNI EF, composite score of executive function in ADNI; ADNI MEM, composite scores of memory in ADNI; MMSE, mini-mental state examination.

Association analysis between psychosis and baseline cognition showed that psychosis was significantly associated with poorer memory function measured by ADNI MEM [*β* = −0.622, *p* = 0.013, odd ratio (OR) = 0.536, 95% confidence interval (CI): 0.323, 0.870] and executive function measured by ADNI EF (*β* = −0.516, *p* = 0.003, OR = 0.596, 95% CI: 0.418, 0.839) (Figure 1). Psychosis was not associated with MMSE score (*β* = 0.037, *p* = 0.584, OR = 1.037, 95% CI: 0.908, 1.187).

In a subset analysis excluding participants with psychosis at baseline, psychosis was significantly associated with poorer baseline ADNI MEM (*β* = −0.638, *p* = 0.012, OR = 0.528, 95% CI: 0.314, 0.864) and ADNI EF scores (*β* = −0.556, *p* = 0.002, OR = 0.573, 95% CI: 0.397, 0.812), whereas the association with MMSE score was not significant (*β* = 0.044, *p* = 0.527, OR = 1.045, 95% CI: 0.912, 1.199) (Supplementary Table 2).

### 3.3 Association of psychosis with baseline AD CSF biomarkers

**Figure 2:**
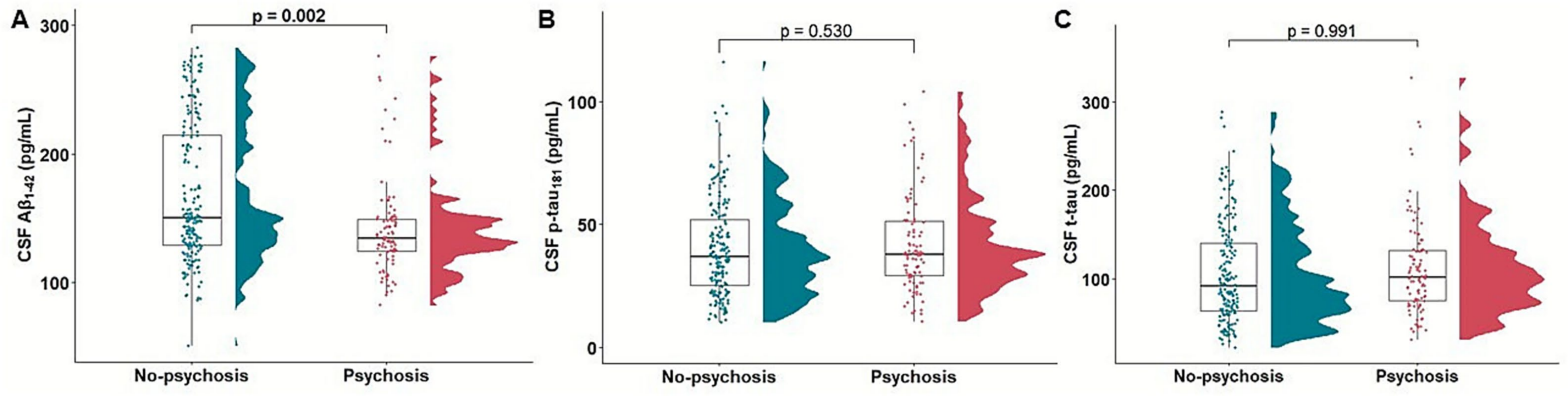
Association of psychosis with baseline CSF Aβ_1-42_, p-tau_181_, and t-tau. Raincloud and boxplots depicting the association of psychosis with baseline CSF Aβ_1-42_ **(A)**, p-tau_181_ **(B)**, and t-tau **(C)**. Aβ, β-amyloid; CSF, cerebrospinal fluid; p-tau, hyperphosphorylated tau; t-tau, total tau.

In association analysis between psychosis and CSF biomarkers of AD pathology, psychosis was significantly related with low CSF Aβ_1-42_ level (*β* = −0.009, *p* = 0.007, OR = 0.990, 95% CI: 0.982, 0.997). However, no significant association was found between psychosis and p-tau_181_ level (*β* = −0.006, *p* = 0.338, OR = 0.993, 95% CI: 0.980, 1.006) or t-tau level (*β* = −0.001, *p* = 0.699, OR = 0.998, 95% CI: 0.993, 1.004) (Figure 2).

In a subset analysis excluding participants with psychosis at baseline, psychosis was significantly associated with low CSF A*β*_1-42_ level (*β* = −0.010, *p* = 0.005, OR = 0.989, 95% CI: 0.982, 0.996), whereas the association with p-tau_181_ level (*β* = −0.005, *p* = 0.426, OR = 0.994, 95% CI: 0.980, 1.007) and t-tau level (*β* = −0.001, *p* = 0.600, OR = 0.998, 95% CI: 0.992, 1.003) were not significant (Supplementary Table 3).

### 3.4 Mediation effect of baseline CSF A*β*_1-42_ level on the association between baseline cognitive function and psychosis

**Figure 3:**
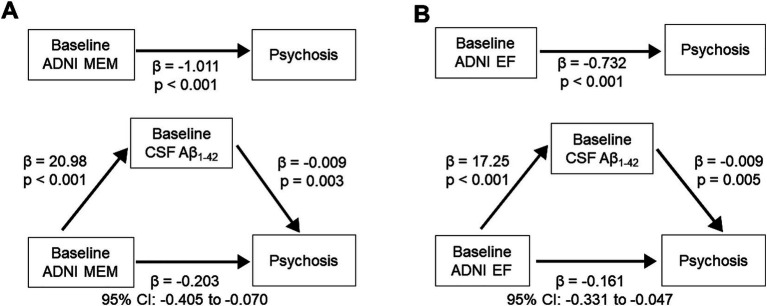
Mediation effect of CSF Aβ_1-42_ level on the association between baseline cognitive function and psychosis. (Top) Direct effect of baseline cognitive function. **(A)** ADNI MEM. **(B)** ADNI EF and psychosis. (Bottom) Mediation effect by baseline CSF Aβ_1-42_ level. Aβ, β-amyloid; ADNI EF, composite score of executive function in ADNI; ADNI MEM, composite scores of memory in ADNI; CSF, cerebrospinal fluid.

Association analyses examining psychosis in relation to cognitive function and CSF biomarkers showed that Psychosis group was associated with poorer baseline memory and executive function, as well as lower baseline CSF A*β*_1-42_ levels. We performed mediation analysis to investigate low CSF A*β*_1-42_ mediates the association between baseline cognitive function and psychosis. The relation between baseline ADNI MEM and psychosis was mediated by low CSF A*β*_1-42_ (*β* = −0.203, 95% CI: −0.405, −0.070) (Figure 3). The relation between baseline ADNI EF and psychosis during AD was also mediated by low CSF Aβ_1-42_ (*β* = −0.161, 95% CI: −0.331, −0.047).

In a subset analysis excluding participants with psychosis at baseline, the relation between baseline ADNI MEM and psychosis was mediated by low CSF Aβ_1-42_ (*β* = −0.201, 95% CI: −0.421, −0.063) (Supplementary Figure S1). The relation between baseline ADNI EF and psychosis during AD was also mediated by low CSF Aβ_1-42_ (*β* = −0.159, 95% CI: −0.345, −0.041).

### 3.5 Association of psychosis with longitudinal changes in CSF AD biomarkers

**Figure 4:**
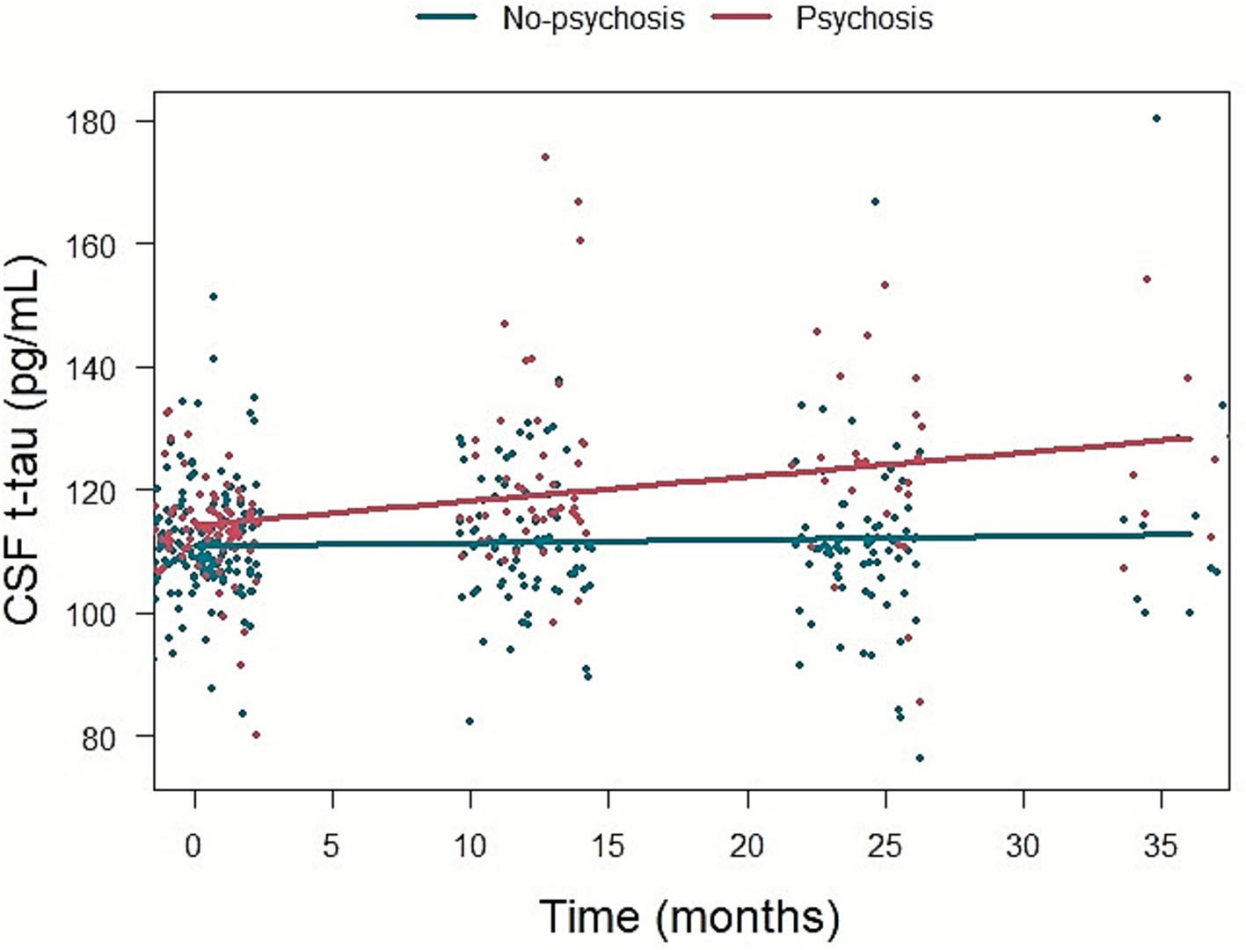
The association of psychosis and longitudinal change in CSF t-tau level. CSF, cerebrospinal fluid; t-tau, total tau.

Psychosis group was associated with faster increase in CSF t-tau level compared to No-psychosis group (*β* = 0.340, *p* = 0.044) (Figure 4). There was no association between psychosis during disease and longitudinal changes in CSF Aβ_1-42_ level (*β* = −0.135, *p* = 0.257) or p-tau_181_ level (*β* = 0.019, *p* = 0.868) (Supplementary Table 4).

In a subset analysis excluding participants with psychosis at baseline, psychosis was significantly associated with a faster increase in CSF t-tau level (*β* = 0.372, *p* = 0.028), whereas the associations with longitudinal changes in CSF Aβ_1-42_ level (*β* = −0.123, *p* = 0.317) and p-tau_181_ level (*β* = 0.007, *p* = 0.947) were not significant (Supplementary Table 5).

## 4 Discussion

In this study we found that psychosis during the disease is associated with poor baseline cognitive function and low baseline CSF Aβ_1-42_ level. We identified the influence of poor baseline cognitive function on presence of psychosis was mediated by low baseline CSF Aβ_1-42_ level. Psychosis during the disease was associated with faster increase in CSF t-tau level.

Association between psychosis and poor cognition has been supported by numerous studies. Poor baseline cognitive function has been known as a predictor of psychosis during the disease in MCI and AD dementia (Paulsen et al., 2000; Weamer et al., 2009; Weamer et al., 2016). There are also many studies presenting AD patients with psychosis showed faster cognitive decline (Ropacki and Jeste, 2005). Even elderly patients without cognitive impairment could present psychosis initially and consequent cognitive decline could follow (Eikelboom et al., 2021). Therefore, poor cognitive function is suggested both as the risk factor of psychosis incidence and as the outcome of psychosis. It is not elucidated that whether underlying pathological mechanism of these two aspects, risk factor and outcome of psychosis, could be on the continuous biological changes or distinctive pathways. In this study, we focused poor cognitive function as a risk factor and found that poor baseline cognitive function was related with psychosis during AD progression, which is aligned with previous studies.

We also found that low baseline CSF Aβ_1-42_ level was related with psychosis during the disease. Association of psychosis and amyloidopathy has been explored in several studies. An autopsy study reported an increased Aβ_1-42_/Aβ_1-40_ concentration ratio in the dorsolateral prefrontal cortex of AD patients with psychosis compared to those without (Murray et al., 2012). Another study also revealed that individual having psychosis without gross cognitive impairment showed higher burden of neuritic plaques compared to those without psychosis (Kim et al., 2018). In amyloid PET-positive MCI patients, higher incidence of hallucination was observed compared with amyloid PET-negative patients (Goukasian et al., 2019).

Based on significant associations of psychosis with poor baseline cognitive function and low baseline CSF Aβ_1-42_ level, we demonstrated the mediating effect of low CSF Aβ_1-42_ level on the relationship between poor baseline cognitive function and psychosis during AD progression. Incidence of psychosis could be merely considered as a result from poor cognitive functions including decline of memory, logical thinking, and disinhibited behavior. However, our finding could show the potential biological link involving amyloidopathy. A recent study reported that the association between baseline psychosis and faster cognitive decline is mediated by a low CSF Aβ_1-42_ level in non-demented elderly individuals (Guo et al., 2024). Although the study focused on a different direction (baseline psychosis–cognitive decline) from ours (baseline cognitive function–psychosis during the disease) and targeted a different population (non-demented elderly vs. MCI and dementia patients), both studies suggested a mediating effect of low CSF Aβ_1-42_ levels on the relationship between cognition and psychosis. Moreover, we demonstrated the relation between psychosis during the disease and faster increase in CSF t-tau level. This could imply psychosis is associated with faster neurodegeneration. Previous study has reported elevated CSF t-tau level and psychosis (Koppel et al., 2013; Satake et al., 2024). In our propensity score–matched cohort with a larger sample size, we found that psychosis during the disease course was associated with lower CSF Aβ_1-42_ levels initially, but a faster increase in t-tau over time.

While our study did not find a significant relationship between psychosis and CSF p-tau_181_ levels, the findings diverge from some previous studies regarding tau pathology. Murray et al. reported elevated phosphorylated tau burden in the prefrontal cortex of AD patients with psychosis compared to those without psychosis (Murray et al., 2014). This discrepancy may be attributable to difference in demographic characteristics of the cohorts. In the study by Murray et al., participants had relatively poor cognitive function, with mean MMSE scores ranging from 9 to 16. In contrast, the mean MMSE score in our cohort was 26. Another study involving amyloid PET–positive patients reported that elevated tau burden on PET was associated with psychosis during the disease course (Gomar et al., 2022). In that study, the group with psychosis had a lower median MMSE score of 20, suggesting a more clinically advanced disease stage. In contrast, the psychosis group in our study may represent an earlier phase of AD. Another study that found a significant association between psychosis and neuritic plaque load—but not with neurofibrillary tangles—included participants with MMSE scores of 26 or higher (Kim et al., 2018). This suggests that the inclusion of participants with predominantly mild disease stages may influence the observed associations. Additionally, methodological differences may have contributed to the discrepancy, particularly the use of CSF biomarkers in our study as opposed to postmortem brain tissue in others. CSF measurements may not fully capture regional heterogeneity in tau pathology. Further research is warranted to elucidate how regional variations in amyloid and tau pathology contribute to psychosis and cognitive dysfunction across AD.

From a clinical perspective, psychosis in AD is influenced by multiple risk factors, including neurotransmitter imbalance, genetic predispositions, as well as environmental and caregiving conditions (Ballard et al., 2020). Moreover, psychosis could present in other neurodegenerative diseases (Naasan et al., 2021). Hence, comprehensive understanding of psychosis presentation, related pathological features, and biomarkers pattern is critical for developing treatment strategies of AD. Our finding may offer insight into the potential mediating role of amyloidopathy in the relationship between cognition and psychosis.

This study has several limitations. First, our reliance on CSF biomarkers rather than direct histopathological analysis limits our ability to assess regional variations in amyloid and tau pathology. Second, psychosis was operationally defined using NPI-Q hallucination and delusion items rather than formal clinical diagnoses. This proxy may not fully capture the clinical complexity of psychotic disorders in AD and may lead to misclassification. Therefore, our findings should be interpreted as associations with psychosis-related symptoms rather than definitive clinical diagnoses.

## 5 Conclusion

Our findings demonstrate that psychosis during AD progression is associated with lower CSF Aβ_1-42_ levels and poorer cognitive function. Mediation analysis further revealed that the association between psychosis and cognitive impairment is partially mediated by reduced CSF Aβ_1-42_, suggesting a potential pathological link between amyloid burden and the manifestation of psychosis in AD. These results underscore the importance of amyloid pathology in the development of psychosis in AD and highlight the need for further investigation into its mechanistic role in cognitive decline.

## Data Availability

Publicly available datasets were analyzed in this study. This data can be found at: https://ida.loni.usc.edu/login.jsp?project=ADNI.

## Footnotes

1
http://www.adni.loni.usc/edu/data-samples/biospecimen-data

## Group members of the Alzheimer’s Disease Neuroimaging Initiative

Data used in preparation of this article were obtained from the Alzheimer’s Disease Neuroimaging Initiative (ADNI) database (adni.loni.usc.edu). As such, the investigators within the ADNI contributed to the design and implementation of ADNI and/or provided data but did not participate in analysis or writing of this report. A complete listing of ADNI investigators can be found at: http://adni.loni.usc.edu/wp-content/uploads/how_to_apply/ADNI_Acknowledgement_List.pdf. Data collection and sharing for this project was funded by the Alzheimer’s Disease Neuroimaging Initiative (ADNI) (National Institutes of Health Grant U01 AG024904) and DOD ADNI (Department of Defense award number W81XWH-12-2-0012). ADNI is funded by the National Institute on Aging, the National Institute of Biomedical Imaging and Bioengineering, and through generous contributions from the following: AbbVie, Alzheimer’s Association; Alzheimer’s Drug Discovery Foundation; Araclon Biotech; BioClinica, Inc.; Biogen; Bristol-Myers Squibb Company; CereSpir, Inc.; Cogstate; Eisai Inc.; Elan Pharmaceuticals, Inc.; Eli Lilly and Company; EuroImmun; F. Hoffmann-La Roche Ltd. and its affiliated company Genentech, Inc.; Fujirebio; GE Healthcare; IXICO Ltd.; Janssen Alzheimer Immunotherapy Research & Development, LLC.; Johnson & Johnson Pharmaceutical Research & Development LLC.; Lumosity; Lundbeck; Merck & Co., Inc.; Meso Scale Diagnostics, LLC.; NeuroRx Research; Neurotrack Technologies; Novartis Pharmaceuticals Corporation; Pfizer Inc.; Piramal Imaging; Servier; Takeda Pharmaceutical Company; and Transition Therapeutics. The Canadian Institutes of Health Research is providing funds to support ADNI clinical sites in Canada. Private sector contributions are facilitated by the Foundation for the National Institutes of Health (www.fnih.org). The grantee organization is the Northern California Institute for Research and Education, and the study is coordinated by the Alzheimer’s Therapeutic Research Institute at the University of Southern California. ADNI data are disseminated by the Laboratory for Neuro Imaging at the University of Southern California.
