# Supplementary material for: Association of psychosis with cognitive impairment is mediated by amyloidopathy in cognitive impairment
Source: Front Aging Neurosci. 2026 Jan 12;17:1663120. doi: 10.3389/fnagi.2025.1663120 (PMC12832791; doi:10.3389/fnagi.2025.1663120)
Supplement: Supplementary file 1 [file Data_Sheet_1.docx]

Supplementary table 1. Demographics of study participants without psychosis at baseline

|  | Psychosis (n = 84) | No-psychosis (n = 192) | *p*-value |
| --- | --- | --- | --- |
| Age | 74.2 ± 7.2 | 75.6 ± 7.9 | 0.183 |
| Female, n (%) | 37 (44.0) | 84 (43.7) | 1.000 |
| Education level | 15.4 ± 2.9 | 15.3 ± 2.9 | 0.843 |
| *APOE* ε4 carrier, n (%) | 54 (64.2) | 95 (49.4) | 0.032 |
| Follow-up duration (months) | 22.1 ± 16.2 | 20.1 ± 16.9 | 0.373 |
| MMSE | 25.4 ± 2.4 | 26.5 ± 2.7 | 0.003 |
| CDR | 0.6 ± 0.2 | 0.5 ± 0.1 | 0.002 |
| CDR SOB | 3.3 ± 1.8 | 2.0 ± 1.6 | < 0.001 |
| ADNI MEM | -0.6 ± 0.6 | -0.1 ± 0.7 | < 0.001 |
| ADNI EF | -0.7 ± 1.0 | -0.07 ± 0.9 | < 0.001 |
| CSF Aβ_1-42_ (pg/mL) | 141.4 ± 38.7 | 168.9 ± 54.0 | < 0.001 |
| CSF p-tau_181_ (pg/mL) | 42.2 ± 19.8 | 40.8 ± 23.2 | 0.612 |
| CSF t-tau (pg/mL) | 110.3 ± 54.2 | 105.5 ± 56.7 | 0.513 |

Data are presented as the mean ± standard deviation or n (%).

Abbreviation: Aβ, β-amyloid; ADNI EF, composite score of executive function in ADNI; ADNI MEM, composite scores of memory in ADNI; CDR, clinical dementia rating; CDR SOB, clinical dementia rating sum of boxes; CSF, cerebrospinal fluid; MMSE, mini-mental state examination; NA: not applicable; p-tau, hyperphosphorylated tau; t-tau, total tau.

Supplementary table 2. Association between psychosis and cognitive function in participants without psychosis at baseline

| Variables | OR (95% CI) | β | *p*-value |
| --- | --- | --- | --- |
| ADNI MEM | 0.528 (0.314, 0.864) | −0.638 | 0.012 |
| ADNI EF | 0.573 (0.397, 0.812) | −0.556 | 0.002 |
| MMSE | 1.045 (0.912, 1.199) | 0.044 | 0.527 |

Age, sex, education, CDR SOB, and *APOE* ε4 carrier status were adjusted.

Abbreviation: ADNI EF, composite score of executive function in ADNI; ADNI MEM, composite scores of memory in ADNI; MMSE, mini-mental state examination; CI, confidence interval; OR, odd ratio.

Supplementary table 3. Association of psychosis with baseline CSF Aβ_1-42_, p-tau_181_, and t-tau in participants without psychosis at baseline

| Variables | OR (95% CI) | β | *p*-value |
| --- | --- | --- | --- |
| CSF Aβ_1-42_ (pg/mL) | 0.989 (0.982, 0.996) | −0.010 | 0.005 |
| CSF p-tau_181_ (pg/mL) | 0.994 (0.980, 1.007) | −0.005 | 0.426 |
| CSF t-tau (pg/mL) | 0.998 (0.992, 1.003) | −0.001 | 0.600 |

Age, sex, CDR SOB, and *APOE* ε4 carrier status were adjusted.

Abbreviation: Aβ, β-amyloid; CI, confidence interval; CSF, cerebrospinal fluid; OR, odd ratio; p-tau, hyperphosphorylated tau; t-tau, total tau.

Supplementary table 4. The association of psychosis with longitudinal CSF AD biomarkers

|  | Psychosis during disease$\times$time | |
| --- | --- | --- |
|  | β | *p*-value |
| CSF Aβ_1-42_ (pg/mL) | -0.135 | 0.257 |
| CSF p-tau_181_ (pg/mL) | 0.019 | 0.868 |
| CSF t-tau (pg/mL) | 0.340 | **0.044** |

Age, sex, and *APOE* ε4 carrier status were adjusted.

Abbreviation: Aβ, β-amyloid; AD, Alzheimer’s disease; CSF, cerebrospinal fluid; p-tau, hyperphosphorylated tau; t-tau, total tau.

Supplementary table 5. The association of psychosis with longitudinal CSF AD biomarkers in participants without psychosis at baseline

|  | Psychosis during disease$\times$time | |
| --- | --- | --- |
|  | β | *p*-value |
| CSF Aβ_1-42_ (pg/mL) | -0.123 | 0.317 |
| CSF p-tau_181_ (pg/mL) | 0.007 | 0.947 |
| CSF t-tau (pg/mL) | 0.372 | **0.028** |

Age, sex, and *APOE* ε4 carrier status were adjusted.

Abbreviation: Aβ, β-amyloid; AD, Alzheimer’s disease; CSF, cerebrospinal fluid; p-tau, hyperphosphorylated tau; t-tau, total tau.
