# Supplementary figures and images for: Association of psychosis with cognitive impairment is mediated by amyloidopathy in cognitive impairment
Source: Front Aging Neurosci. 2026 Jan 12;17:1663120. doi: 10.3389/fnagi.2025.1663120 (PMC12832791; doi:10.3389/fnagi.2025.1663120)

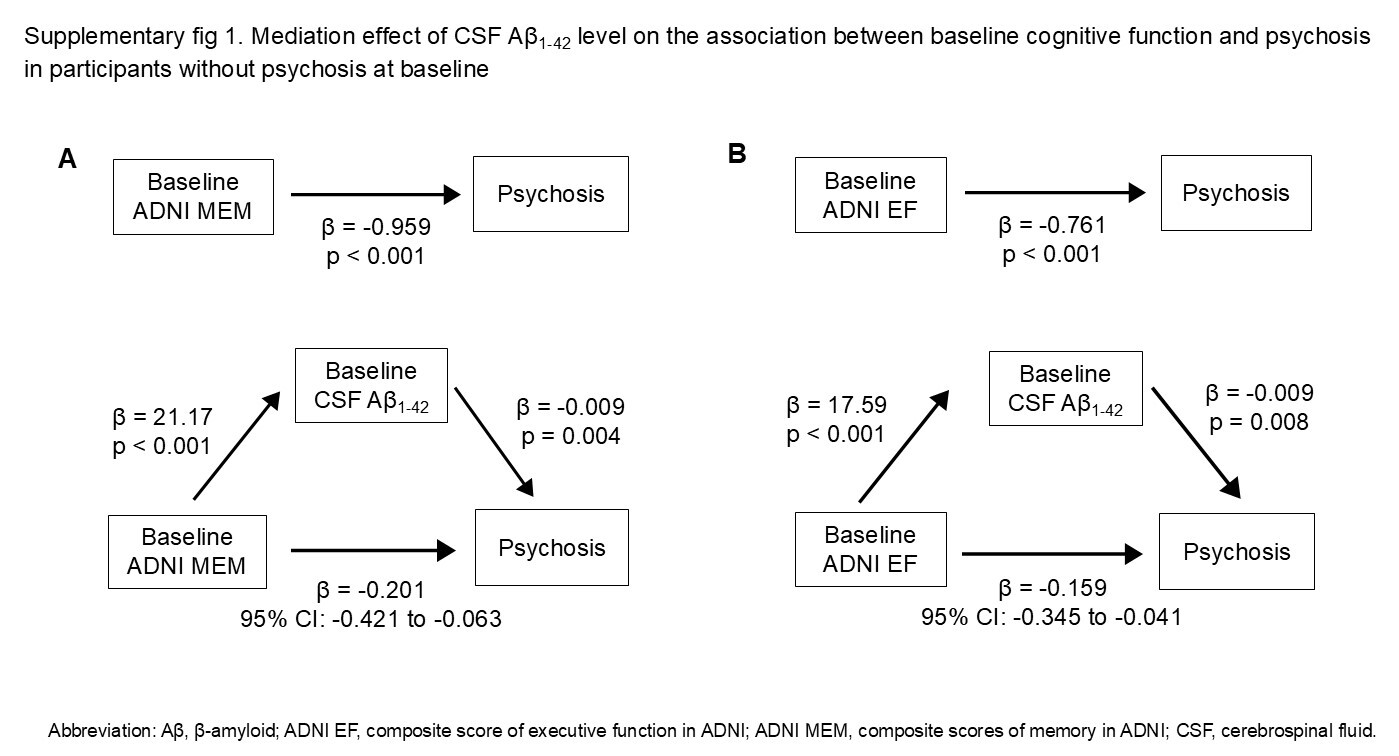

Supplement: Supplementary file 2 [file Image_1.jpg]
